# The Utstein template for uniform reporting of data following major trauma: A valuable tool for establishing a pan-European dataset

**DOI:** 10.1186/1757-7241-16-8

**Published:** 2008-08-28

**Authors:** Karim Brohi

**Affiliations:** 1 Trauma and Vascular Surgery, Barts and the London NHS Trust, London, UK

## 

Trauma is a public health problem of global importance. Central to any public health approach in tackling a disease is research, and central to any research is high quality data. Trauma systems – local, regional, national or international – are driven by the completeness and quality of data they collect and analyze. The performance improvement cycle of system assessment, policy development and quality assurance is dependent on the informatics infrastructure that supports it. Solid, quality data is a powerful tool in improving care, for clinicians, administrators and politicians.

Trauma registries are therefore key elements of any public health approach to managing severe injury. The past two decades have seen several European countries recognise the importance of this and successfully develop their own national registries. Each country continues to face its own difficulties with regard to completeness, data quality and funding for informatics management. Nevertheless, a recent publication by Ringdal et al [1] have recognised the enticing potential of a pan-European registry. The value of these datasets increases almost exponentially with the amount of data collected. Beyond simple size implications, a pan-European dataset will allow comparison of different methods of delivering trauma care, Europe-wide programmes for clinical research and large-scale funding and policy development. Reminiscent of the spirit of the European Union the authors have not attempted to foist a single tool on all members, but rather attempted to agree a core dataset common to all which can be built on to respond to the specific needs of individual states.

The Utstein Group have attempted to reduce the core dataset from 92 parameters to only 35. There are rational arguments for doing so, and a smaller dataset is more likely to be collected in its entirety. Some datapoints are redundant, and coupled so closely to others that their utility is minimal. Some datapoints provide no information on outcomes, system characteristics or process of care. Some are superseded by newer measures or tools. Both the reasoning behind the reduction in parameters, and the methodology by which the Utstein participants have proceeded are robust, and the resultant dataset is interesting for its conciseness in what it contains – and in what has been left out.

But is this reductionist approach the right one for our times? Can this 35-point dataset tell us not only that outcomes are different in different systems but why? Will they allow rational trial design? Will they ensure that funding is targeted to the right regions and system components? More than likely they will provide a simplistic overview, which may be suggestive, and may be wrong. Making sensible decisions about the delivery of care, outcomes after severe injury or planning clinical trials requires more, not less data. And not a little but more but tens or hundreds of times more data items than we currently have. It is a conceit to think that a simple scoring system for injury severity, and a single-time point assessment of physiology can really reflect the true state of a patient on arrival. Trauma is a dynamic process – patients may be improving or deteriorating on arrival. Standard registry data pays no attention to this and little attention to the response to resuscitative manoeuvres. And while functional outcomes are harder and more costly to measure, they must be more valuable and descriptive of patient injuries and the care they received.

Much of this data can now be collected automatically, given the appropriate equipment and infrastructure. Prehospital data can be downloaded wirelessly from a paramedic's PDA as soon as they come within wireless range of the admitting hospital. Physiological data, drug and blood product administration can all be tagged electronically, as can the patient's position within the hospital. Other databases, such as ICU, pathology and radiology systems already collect much of the diagnostics, morbidity and outcome data on these patients. We have enough computer processing power and data storage for these needs. New statistical and data-mining techniques allow for the combination of such databases, the management of missing data and the sensible exploration of large-scale data.

For now, both approaches are valid. A robust, clearly defined core dataset common to all will be a very valuable tool. It will be interesting to see whether this smaller dataset will allow more complete data collection on larger numbers of patients, and whether valuable analyses can be derived from it. Important for this will be legislative policy at national and European levels to support the development of an informatics infrastructure for trauma and the collection of such data on a population-wide basis. However, we must rapidly move towards a whole-system approach to data collection, for it is only in the complexity of our trauma patients, and the multitude of interventions they are exposed to, that the true future of trauma care resides.

## Competing interests

The author declares that they have no competing interests.
